# Concomitant ablation for non-paroxysmal atrial fibrillation: combined energy versus cryoablation alone

**DOI:** 10.3389/fcvm.2024.1448523

**Published:** 2024-09-18

**Authors:** Bashir Tsaroev, Ravil Sharifulin, Alexander Afanasyev, Sergey Khrushchev, Murtazali Murtazaliev, Darya Lovtsova, Robert Kashapov, Pavel Ruzankin, Muslim Mustaev, Alexander Bogachev-Prokophiev

**Affiliations:** ^1^ Department of Adult Cardiac Surgery, E.N. Meshalkin National Medical Research Center, Novosibirsk, Russia; ^2^ Laboratory of Applied Inverse Problems, Sobolev Institute of Mathematics, Novosibirsk, Russia; ^3^ Department of Mathematics and Mechanics, Novosibirsk State University, Novosibirsk, Russia; ^4^ Department of Adult Cardiac Surgery, Guy’s and St Thomas’ NHS Foundation Trust, London, United Kingdom

**Keywords:** atrial fibrillation, maze procedure, biatrial ablation, concomitant ablation, radiofrequency ablation, cryoablation

## Abstract

**Background:**

Surgical ablation of atrial fibrillation has been the most efficient treatment for atrial fibrillation (AF). Combined energy (CE) ablation and cryoablation alone (CA) are the most common energy modes used for ablation, however, comparative data is lacking.

**Objectives:**

To compare the efficacy of CE ablation with CA in the setting of concomitant biatrial ablation for non-paroxysmal AF.

**Methods:**

A retrospective analysis of 453 patients with non-paroxysmal AF undergone concomitant biatrial ablation from November 2007 to December 2022 during elective cardiac surgery using either combined bipolar radiofrequency with cryoenergy or cryoenergy alone was performed. Propensity score matching was conducted to balance the covariates in the groups.

**Results:**

There were 157 patients per group after matching. CE ablation was associated with lower odds of atrial tachyarrhythmia recurrence (OR = 0.13, 95% CI 0.02–0.91, *p* = 0.040), a significantly lower rate of hospital readmissions due to rhythm disruption (HR = 0.34, 95% CI 0.18–0.65, *p* < 0.001), and lower cumulative incidence of stroke (SHR = 0.38, 95% CI 0.15–0.97, *p* = 0.043). No significant difference in permanent pacemaker implantation was observed between the two groups.

**Conclusions:**

In the setting of concomitant biatrial ablation for non-paroxysmal AF, combined bipolar radiofrequency and cryoablation appear to be a superior treatment modality compared to cryoablation alone in achieving long-term freedom from atrial arrhythmias, in reducing arrhythmia-related hospital readmissions and ischemic strokes.

## 1 Introduction

The Maze procedure, introduced by J. Cox in 1987, is proven to be the most effective treatment option for patients with symptomatic atrial fibrillation (AF). Initially utilized for lone AF, the procedure gained a broader application with the advent of cryoablation in 1997, and then combined radiofrequency and cryoablation in 2002, particularly in treating concomitant AF (1, 2).

Nowadays, the primary energy sources employed for ablating the atrial tissue are bipolar radiofrequency and cryoenergy, among many others. When radiofrequency energy is used, cryoablation is usually utilized to create lesions towards the atrioventricular valves, leading to its designation as combined energy (CE) ablation.

Although the cryo-Maze procedure is a durable treatment option used in many units worldwide, comparative data on efficacy of each energy source is lacking. Hence, the objective of this study was to compare these two energy modalities in the context of concomitant biatrial ablation in patients with non-paroxysmal AF and evaluate the outcomes of each approach.

## 2 Methods

This study constitutes a retrospective analysis of a single-center experience. Preoperative demographic data, operative details, and perioperative results were extracted from our institutional database. Approval for the study was granted by the institutional review board of the E.N. Meshalkin National Medical Research Center, under the protocol #14 dated as at September 26, 2022. Informed consent was waived as this was a retrospective analysis of the anonymized data.

### 2.1 Study population

All patients who underwent biatrial ablation for non-paroxysmal AF during elective cardiac surgery since the start of the AF ablation program at our center in November 2007 were included into the study. Two energy sources were exclusively employed, either bipolar radiofrequency or cryoablation, by which two comparative groups were formed, CE ablation and CA groups. The decision to opt in for either CE ablation or cryoablation alone (CA) was at the discretion of the operating surgeon. The elective cardiac surgery procedures included various operations, such as valve surgery, coronary artery bypass grafting (CABG), septal myectomy, and others.

### 2.2 Surgical technique

**Figure 1:**
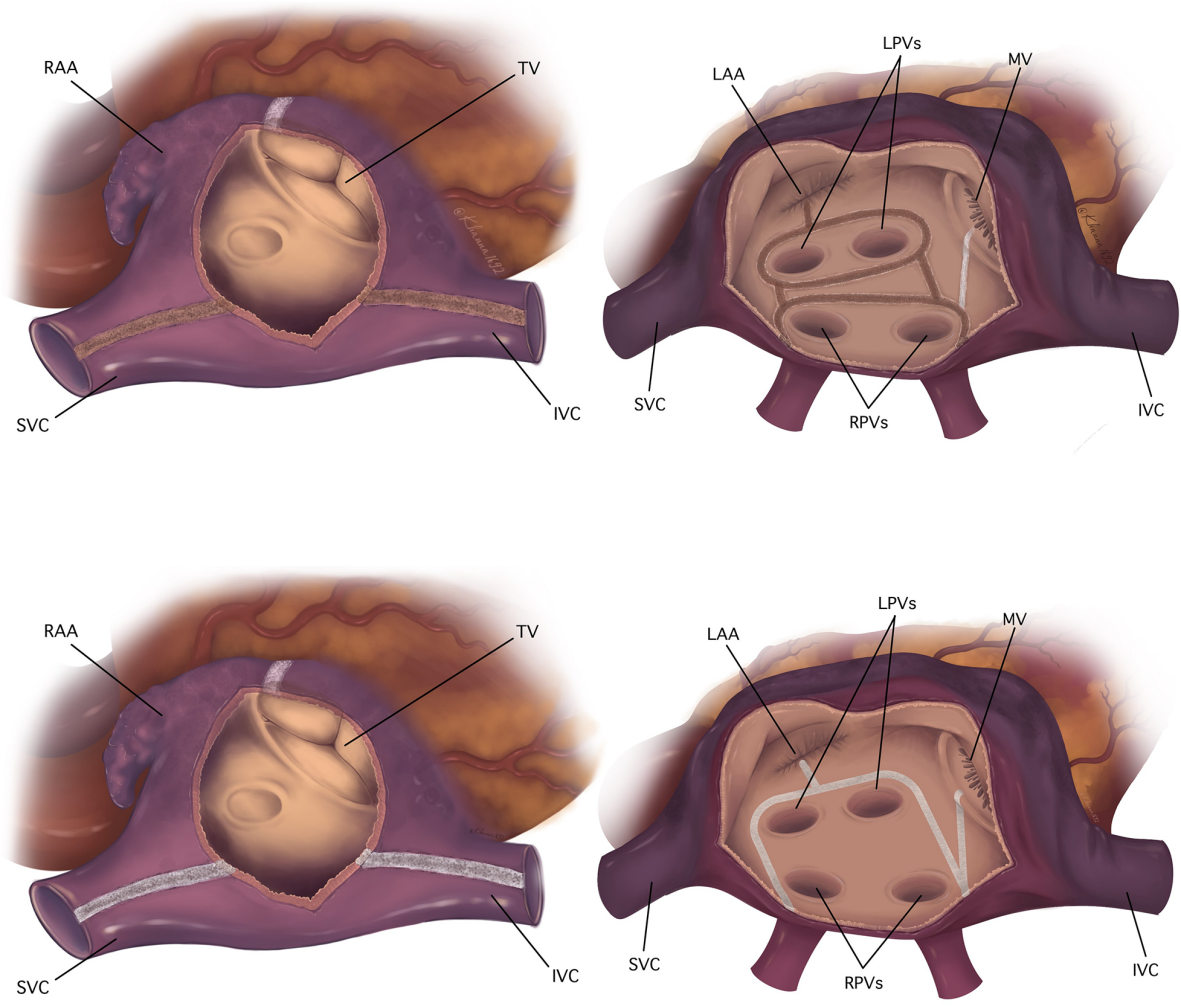
Biatrial pattern of lesions utilized in the studied population. The top: the ablation scheme used in combined energy group, the bottom: the ablation scheme used in cryoablation alone group. The right atrial lesion set is on the left side; the left atrial lesions are on the right side. Radiofrequency lesions are colored brown, cryolesions are colored white. IVC, inferior vena cava; LAA, left atrial appendage; LPVs, left pulmonary veins; MV, mitral valve; RAA, right atrial appendage; RPVs, right pulmonary veins; SVC, superior vena cava; TV, tricuspid valve.

The lesion set utilized in this study was described in our previous articles and is illustrated in Figure 1 (3, 4). Here are some technical nuances of each approach. The radiofrequency clamp was applied 8–10 times for one lesion. The cryoprobe was administered for two minutes once from endocardial side for each lesion; the 2-min count started when the probe temperature reached −60°C. The lesion to the mitral valve was further supplemented by epicardial application of the cryoprobe towards the coronary sinus for two minutes. The cryoprobe was applied on the arrested heart only, while for the bipolar radiofrequency clamp the arrested heart was not required. The patterns of the lesions in non-mitral valve surgery were identical to those in mitral valve operations: for instance, the ablative lesions were applied through the opened left atrium, and the same lesion sets were maintained throughout the both groups. The left atrial appendage was excised or excluded using endocardial or epicardial suture.

### 2.3 Follow-up and definitions

Anticoagulation therapy and antiarrhythmic drugs were initiated in all patients soon after surgery, normally from the post-operative day 1 and onwards, unless contraindicated. Electrical cardioversion was employed if antiarrhythmics were proved ineffective in preventing atrial tachyarrhythmia (ATA) during the hospital stay. The duration of anticoagulation therapy and/or antiarrhythmics was reviewed at three months post-operatively during a follow-up visit. The decision was made based on the clinical guidelines, patients' history, the results of 24-h Holter monitoring and patient assessment in the clinic. Subsequent adjustments to anticoagulation or antiarrhythmic therapy were made by a local cardiologist.

All patients were advised to attend a follow-up visit in our center at 3, 6, and 12 months, followed by the annual appointments. In the cases where patients failed to attend the follow-up visit, telephone interviews were conducted, and relevant medical documentation was obtained via email.

Sinus rhythm disruption or ATA recurrence was defined as an episode of AF, atrial flutter, or atrial tachyarrhythmia lasting longer than 30 s and these were registered utilizing an ECG, Holter monitoring, or medical records from other hospitals. Sinus rhythm recorded exclusively by 24-h or 48-h ECG monitoring was used for the analysis. The first three months after the operation were considered a blanking period during which the rhythm outcomes were not recorded.

Rehospitalizations due to disruption of sinus rhythm or ATA-related readmissions were defined as any hospitalization requiring intervention (cardioversion or catheter ablation) to restore sinus rhythm, with at least one overnight stay. Permanent pacemaker (PPM) implantation after discharge was analyzed separately from PPM implantation during the index hospital admission. The analysis of stroke was focused on freedom from ischemic strokes only, as hemorrhagic stroke could not be directly attributed to atrial tachyarrhythmias, with only one known case in this category.

### 2.4 Statistical analysis

Data analysis and visualization were conducted using R software, version 4.2.3. Continuous variables were presented as median with interquartile range, while categorical data were expressed as absolute numbers and relative frequencies. The comparison of unmatched groups utilized the Mann–Whitney *U*-test for continuous variables, and χ^2^ test or the Fisher exact test, as appropriate, for categorical variables. Continuous variables were compared between matched groups using the Wilcoxon signed-rank test for paired samples, and binary variables were compared between matched groups using univariable conditional logistic regression. Following the guidelines on reporting data in AF studies, we utilized mixed effects logistic regression models for assessment of 24-h Holter monitoring data and competing risks models for assessment of long-term freedom from PPM implantation and ischemic strokes (5). We used a doubly robust approach, applying multivariate analysis to the matched groups.

The primary outcome was atrial fibrillation or atrial flutter or atrial tachyarrhythmia lasting longer than 30 s observed at least once in the corresponding year. The patient was considered as a random effect in the model. The covariates for the models are listed in Table 1 where the results of the univariate models and the multivariable model are reported. The multivariable model included all the listed covariates. Patients lacking rhythm follow-up data after the blanking period were excluded from the analysis. For the analysis, the rhythm data were truncated at 7 years, because beyond this period, no sufficient Holter data were available for comprehensive assessment.

**Table 1:** Predictors of ATAs reoccurrence during follow-up (mixed effects logistic regression model).

Predictors	Multivariate analysis		
	OR	95% CI	*p*-value
Combined energy ablation	0.13	0.02–0.91	**0.040**
Years since operation	1.41	1.13–1.77	**0.003**
ATA occurrence in hospital	22.04	7.49–69.84	**0.002**
In-hospital cardioversion	0.17	0.01–4.58	0.294
Atrial fibrillation duration	1.07	0.91–1.26	0.421
Mitral valve replacement	1.33	0.21–8.43	0.764
Aortic valve replacement	0.10	0.01–1.04	0.054
Coronary artery bypass grafting	4.01	0.28–58.32	0.309

ATA, atrial tachyarrhythmias; CI, confidence interval; OR, odds ratio.

Bold values indicate the *p*-values <0.05.

Recurrent event analysis was applied to analyze hospital readmissions due to ATA (6). In this study, we used the term “hazard ratio” (HR), and not the “rate ratio” for recurrent events because HR is clearer for general audience and the rate ratio for recurrent events bears the same meaning as does the hazard ratio for terminating events. For the analysis of recurrent events, we used the model presented in (Huang and Huang) with the Gehan weight function, implemented in the reReg R package (7). Following the guidelines, we utilized competing risks models for assessment of long-term freedom from permanent pacemaker implantation and ischemic strokes, death being considered the competing risk in the both cases (5).

Propensity score matching (PSM) was implemented to simulate randomization to improve covariate balance. The propensity score was estimated with a logistic model using the following covariates: age, sex, left ventricular ejection fraction, left atrial size, previous catheter ablation, body mass index, hypertension, diabetes mellitus, chronic obstructive pulmonary disease, ischemic heart disease, degenerative or rheumatic heart valve disease, infective endocarditis, hypertrophic cardiomyopathy, congenital heart disease, previous stroke and transient ischemic attack, peripheral vascular disease. One-to-one matching was performed by greedy nearest neighbor matching without replacement. The caliper width 0.1 was applied to the score for the matching. To check the balance between the groups after matching, absolute standardized differences were calculated as absolute standardized differences of means for continuous variables and as absolute differences of proportions for binary variables. Additionally, for continuous variables, the variance ratios were calculated. A variable was considered balanced between the groups if the absolute standardized difference was <0.1 and, in the case of continuous variables, the variance ratio was <2 and >0.5.

## 3 Results

There were 453 patients identified and allocated to two groups before matching – CE group (*N* = 191) and CA group (*N* = 262) before PSM. The median age was 59 years with younger patients being in the CE group, and there were more female patients in CA group. Patients with degenerative heart valve disease prevailed in CA group, while in CE group there were more patients with rheumatic heart valve disease. As a consequence, mitral valve replacement was performed more often in the latter group. After PSM two groups were balanced and 157 patients per group were allocated. The demographic and perioperative characteristics of studied population before after matching are presented in Supplementary Table S1, while demographic and perioperative data after matching are presented in Table 2.

**Table 2:** Preoperative data.

Characteristic	Cryoablation alone; *N* = 157	Combined energy ablation; *N* = 157	*p*-value	*ASD*
Age, years	59 (50, 64)	57 (50, 64)	0.371	0.089
Sex, male	90 (57%)	92 (59%)	0.820	0.013
Atrial fibrillation duration, years	3.0 (1.0, 6.0)	3.0 (1.0, 6.0)	0.400	0.041
Left ventricular ejection fraction, %	57 (51, 63)	58 (50, 64)	0.713	0.035
Left atrium size^a^, cm	6.60 (6.20, 7.30)	6.60 (6.10, 7.30)	0.891	0.026
Previous catheter ablation	3 (1.9%)	2 (1.3%)	0.662	0.006
Previous myocardial infarction	14 (8.9%)	10 (6.4%)	0.421	0.025
Body mass index	28.0 (24.1, 32.0)	28.4 (25.5, 31.6)	0.781	0.03
Hypertension	77 (49%)	76 (48%)	0.901	0.006
Diabetes mellitus	21 (13%)	18 (11%)	0.590	0.019
Degenerative heart valve disease	54 (34%)	48 (31%)	0.450	0.038
Rheumatic heart valve disease	88 (56%)	96 (61%)	0.312	0.051
Ischemic heart disease	33 (21%)	30 (19%)	0.702	0.019
Hypertrophic obstructive cardiomyopathy	5 (3.2%)	6 (3.8%)	0.76	0.006
Infective endocarditis	8 (5.1%)	6 (3.8%)	0.603	0.013
Peripheral vascular disease^b^	41 (26%)	41 (26%)	>0.99	0
Chronic obstructive pulmonary disease	9 (5.7%)	7 (4.5%)	0.621	0.013
Previous stroke	11 (7.0%)	9 (5.7%)	0.643	0.013
Previous transient ischemic attack	3 (1.9%)	1 (0.6%)	0.340	0.013
Time in operating room, min	258 (210, 315)	253 (220, 319)	0.600	0.009
Cross-clamp time, min	100 (83, 125)	107 (87, 129)	0.301	0.041
Mitral valve replacement	86 (55%)	95 (61%)	0.312	0.057
Aortic valve replacement	31 (20%)	33 (21%)	0.771	0.013
CABG	21 (13%)	26 (17%)	0.462	0.032
ATA recurrence in hospital	69 (44%)	53 (34%)	0.069	0.102
Electrical cardioversion	34 (22%)	38 (24%)	0.626	0.025
Hospital mortality	3 (1.9%)	5 (3.2%)	0.481	0.013
Perioperative myocardial infarction	3 (1.9%)	2 (1.3%)	0.663	0.006
In-hospital stroke	2 (1.3%)	1 (0.6%)	0.572	0.006
PPM implantation in hospital	11 (7%)	17 (11%)	0.263	0.038
ATA at discharge	31 (20%	27 (17%)	0.583	0.027
Left ventricular ejection fraction at discharge, %	56 (50, 61)	58 (49, 65)	0.141	0.146

Continuous variables are presented as median with interquartile range. Categorical data are expressed as absolute numbers and relative frequencies.

ATA, atrial tachyarrhythmias; CABG, coronary artery bypass grafting; PPM, permanent pacemaker, ASD, absolute standardized difference.

^a^ Long axis view on transthoracic echocardiography.

^b^ Hemodynamically significant.

### 3.1 Atrial tachyarrhythmia reoccurrence

**Figure 2:**
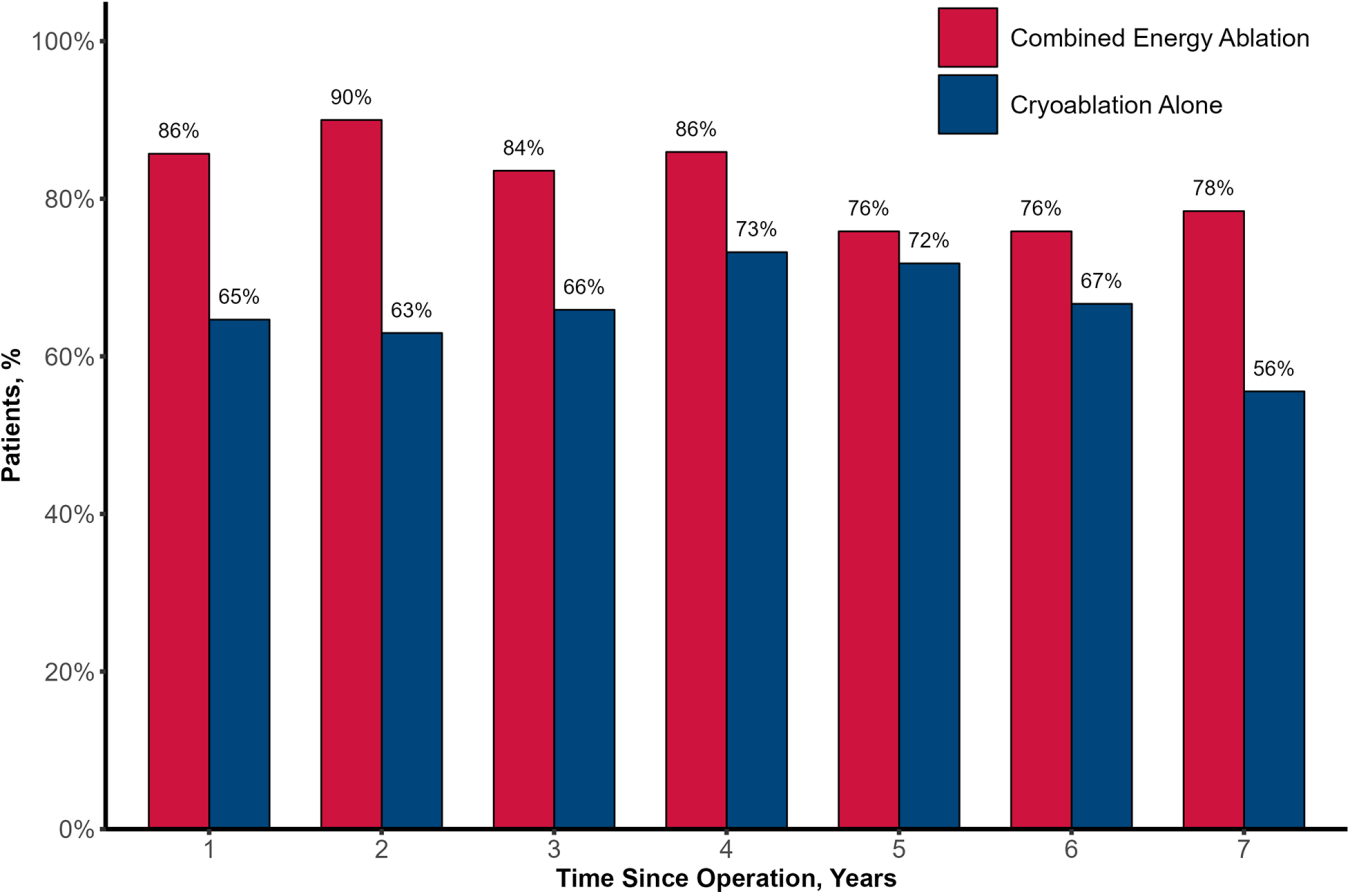
Freedom from atrial tachyarrhythmia. Proportion of patients in combined energy ablation and cryoablation alone groups free from atrial tachyarrhythmia each year over 7 years of follow-up.

Sinus rhythm restoration exhibited a consistent descending pattern throughout the follow-up, with higher proportions of the restoration observed in the CE group each year. The numbers of patients in CE group free from ATA in the 1st and 7th year were 86% and 78%, respectively, and in CA group were 65% and 58%, respectively (see Figure 2). Mixed effects logistic regression model was employed in order to identify potential predictors of long-term sinus rhythm disruption (Table 1). The use of bipolar radiofrequency energy was associated with a statistically significant decrease in the odds of long-term sinus rhythm disruption (odds ratio [OR] = 0.13, 95% confidence interval [CI] 0.02–0.91, *p* = 0.031). Moreover, the odds of long-term ATA reoccurrence demonstrated a significant increase over time, and experiencing AF or atrial flutter event during the hospital stay was associated with an increase in the odds of long-term loss of sinus rhythm (Table 1).

### 3.2 Rehospitalization due to sinus rhythm disruption

**Figure 3:**
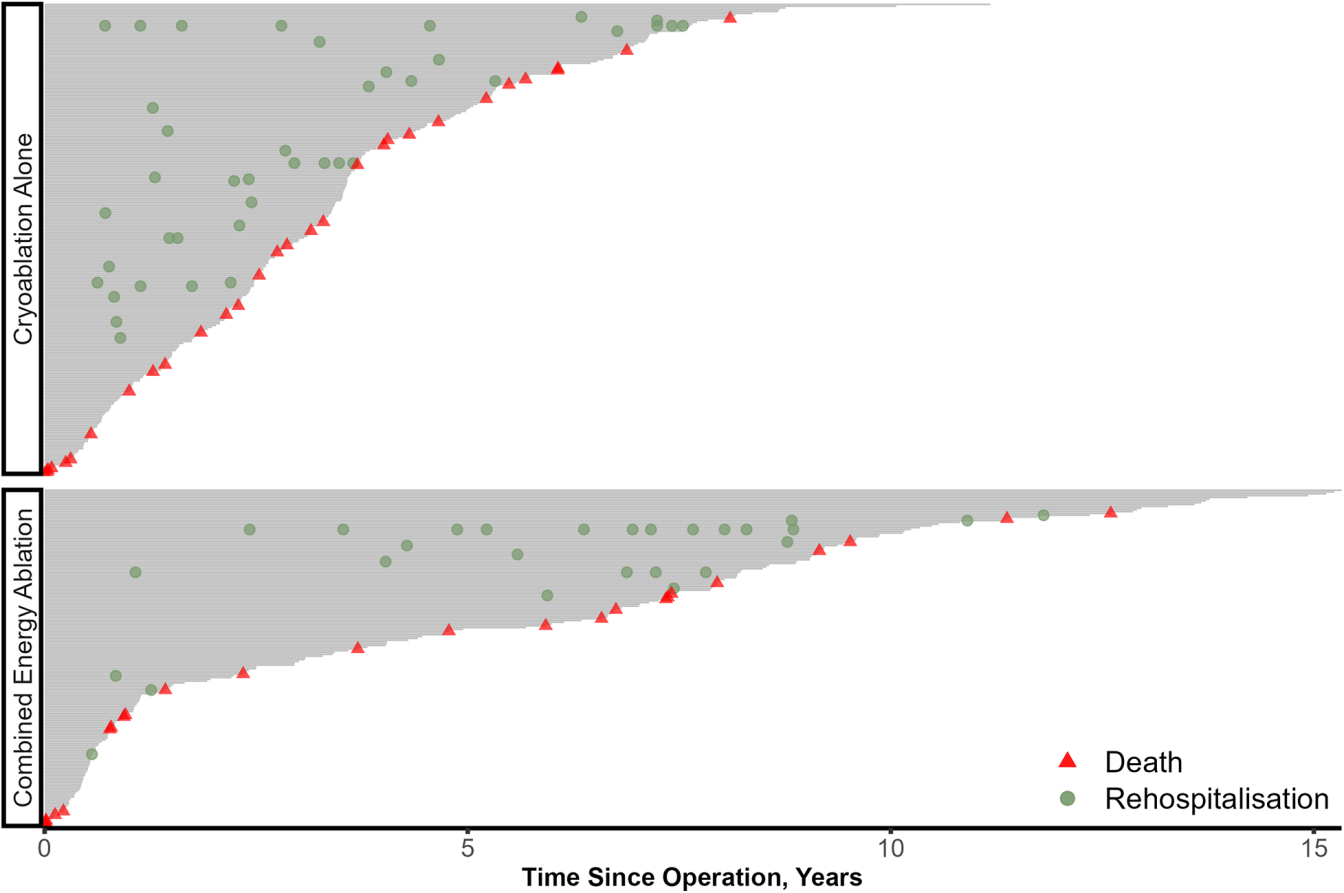
Rehospitalizations due to sinus rhythm disturbance. Each patient's follow up is represented as a grey line with rehospitalizations occurring throughout the line.

There were 67 documented rehospitalizations due to sinus rhythm disruption during the follow-up period. In Figure 3, the rate of rehospitalizations requiring sinus rhythm restoration was higher in CA group and the rehospitalizations occurred earlier after discharge. In regression models combined radiofrequency and cryoablation significantly lowered the rate of recurrent rehospitalization, HR = 0.34 (95% CI 0.18–0.65, *p* = 0.001. Other factors that were found to be statistically significant in multivariable analysis included CABG (HR 2.14, 95% CI 1.00–4.57, *p* = 0.049), and in-hospital ATA occurrence (HR 0.21, 95% CI 0.05–0.92, *p* = 0.038). Details of recurrent readmissions analysis are presented in Table 3.

**Table 3:** Recurrent event analysis of ATA-related readmissions (recurrent events regression model).

Characteristic	Multivariate analysis		
	HR	95% CI	*p*-value
Combined energy ablation	0.34	0.18–0.65	**0** **.** **001**
ATA occurrence in hospital	0.21	0.05–0.92	**0**.**038**
In-hospital cardioversion	2.27	0.49–10.65	0.30
Coronary artery bypass grafting	2.14	1–4.57	**0**.**049**
Mitral valve replacement	1.01	0.54–1.87	0.99
Aortic valve replacement	0.65	0.29–1.46	0.29

ATA, atrial tachyarrhythmia; CI, confidence interval; HR, hazard ratio.

Bold values indicate the *p*-values <0.05.

### 3.3 Permanent pacemaker implantation

During follow-up period there were 11 PPM implantations in CA group and 14 in CE cohort. After matching there were 5 PPM implantations in CA group, 10 implantations in CE group. All PPM were implanted for sinus node dysfunction. Clinically relevant perioperative characteristics were evaluated using Fine-Gray regression in order to identify predictors of PPM implantation during the follow-up. In multivariate Fine-Gray model, in-hospital ATA paroxysms [subdistribution hazard ratio (SHR) = 4.26, 95% CI 1.11–16.33, *p* = 0.034] was the only factor found to influence the freedom from PPM implantation after discharge (Table 4). But energy source had no influence either in univariate or multivariable analysis.

**Table 4:** Predictors of permanent pacemaker implantation over follow-up (fine-gray regression model).

Predictors	Multivariate analysis		
	SHR	95% CI	*p*-value
Combined energy ablation	2.35	0.69–7.96	0.17
ATA occurrence in hospital	4.26	1.11–16.33	**0**.**034**
In-hospital cardioversion	0.48	0.12–1.99	0.32
Mitral valve replacement	1.12	0.37–3.35	0.84
Aortic valve replacement	0.51	0.12–2.28	0.38
Coronary artery bypass grafting	1.67	0.6–4.66	0.33
Previous myocardial infarction	1.87	0.42–8.39	0.41

ATA, atrial tachyarrhythmia; CI, confidence interval; SHR, subdistribution hazard ratio.

Bold values indicate the *p*-values <0.05.

### 3.4 Ischemic stroke

There were 39 primary ischemic strokes after discharge and one case of hemorrhagic stroke resulting in death. After matching there were 8 ischemic strokes in CE group, and 12 in CA group. Various clinically relevant perioperative characteristics were assessed using Fine-Gray regression models in order to identify predictors of ischemic stroke occurrence during the follow-up. Factors influencing the incidence of ischemic stroke were the use of CE ablation (SHR = 0.38, 95% CI 0.15–0.97, *p* = 0.043) and mitral valve replacement (SHR = 4.65, 95% CI 1.36–15.86, *p* = 0.014). Other details of the Fine-Gray models are provided in Table 5.

**Table 5:** Predictors of ischemic stroke during follow-up (fine-gray regression models).

Predictors	Multivariate analysis		
	SHR	95% CI	*p*-value
Combined energy ablation	0.38	0.15–0.97	**0**.**043**
ATA occurrence in hospital	0.99	0.41–2.41	0.98
Coronary artery bypass grafting	1.73	0.55–5.42	0.35
Aortic valve replacement	1.32	0.46–3.75	0.61
Mitral valve replacement	4.65	1.36–15.86	**0**.**014**
Left atrial appendage closure	3.80	0.48–30.31	0.21

ATA, atrial tachyarrhythmia; CI, confidence interval; SHR, subdistribution hazard ratio.

Bold values indicate the *p*-values <0.05.

## 4 Discussion

The key finding of this study is superiority of CE ablation over CA for long-term restoration of sinus rhythm, freedom from recurrent hospitalizations due to sinus rhythm disruption and lower incidence of ischemic strokes. Several advantages of bipolar radiofrequency ablation likely contributed to the superiority of CE ablation over CA (8). Firstly, the bipolar clamp delivers energy from both epicardial and endocardial sides, minimizing atrial tissue folding and thereby preventing formation of non-transmural lesions. Secondly, multiple applications with bipolar radiofrequency clamp ensure the transmurality of the lesions. Additionally, a real-time confirmation of lesion depth through tissue impedance measurements allows for delivery of adequate energy level.

In CA group the posterior wall ablation scheme was consisted of only the “box” lesion, while CE group had additionally bilateral pulmonary veins isolation. The difference in the left atrial posterior wall ablation between CA and CE groups could potentially confound the study results. While both ablation schemes are identical from electrophysiological perspective, there is still a possibility of having non-transmural gaps within the each “box” set of lesions which, in case of CE, are potentially offset by bilateral pulmonary vein isolation, thereby reducing the chance of ATA recurrence. However, further electrophysiological mapping studies are needed to prove this concept.

Cryoablation is considered to be more nuanced and operator-dependent procedure than bipolar radiofrequency ablation. Several authors proposed different tips and tricks to reduce the possibility of gaps in atrial tissue such as by freezing the atrial tissue for three minutes instead of commonly used two minutes, by freezing the segments not longer than 5 cm, etc. (9, 10). In some instances, such as significant atrial thickening in patients with hypertrophic cardiomyopathy, endocardial lesions are reinforced with epicardial application of the cryoprobe in order to ensure transmurality of the lesions (11). Also, applying the cryoprobe on the normothermic heart is associated with lower efficacy of cryoablation. In the light of the mentioned above nuances of cryoablation, bipolar radiofrequency ablation appears to be a more reproducible and fool-proof application technique.

Tracking the status of sinus rhythm restoration in our study, with or without antiarrhythmics, posed challenges in the long term for several reasons. The rhythm follow-up data, as mentioned earlier, were primarily collected retrospectively, and the patients faced difficulties recalling previous antiarrhythmic therapy accurately or providing detailed medical records. Besides this, some of the patients were taking medications with antiarrhythmic properties for other cardiac conditions, e.g., beta-blockers for hypertension or heart failure. Still, we categorized our results on restoration of sinus rhythm without antiarrhythmics, as the patients with restored sinus rhythm in the long term did not take agents with antiarrhythmic properties primarily for the treatment or prevention of ATA recurrence. Of note, patients in sinus rhythm ceased their medications several days before Holter monitoring. Similar challenges were encountered in tracking anticoagulation therapy at each time point.

Hospital readmissions related to ATA reflect a subgroup of individuals with symptomatic AF. The odds of such outcomes were significantly lower in CE group, conforming to the results of rhythm analysis. This relationship is particularly important considering the inherent limitations of the intermittent analysis for ATA. Other risk factors for recurrent hospitalizations were female sex, age, and in-hospital ATA occurrence. Interestingly, in-hospital ATA events after surgery tended to lower the rate of rehospitalization which may be explained by more aggressive antiarrhythmic therapy or because such patients tend to remain in AF for longer, and AF eventually becomes permanent.

An inherent drawback of the Maze procedure is the increased risk of PPM implantation, as was demonstrated in several meta-analyses (12, 13). In the study by Li H. et al., biatrial ablation was associated with a higher risk of PPM implantation than left-sided atrial ablation (14). However, the data comparing the two approaches, combined and cryoablation alone, is lacking. Khiabani A. et al. reported the following predictors of PPM implantation: age, female sex, sternotomy, biatrial ablation, and in-hospital ATA events (15). In the competing risks model in our study, the risk factor for PPM insertion were in-hospital paroxysms of ATAs which, in our view, may be associated with more aggressive antiarrhythmic therapy in those patients.

In the current study, the use of CE ablation significantly decreased the SHR in multivariable analysis while mitral valve replacement increased the hazard of ischemic stroke. Assessing the risk of stroke after surgical ablation represents a research challenge. There are numerous confounders such as left atrial appendage occlusion, recanalization of left atrial appendage after occlusion, adherence to anticoagulation therapy and thorough INR control, type of prosthetic valve, left ventricular ejection fraction, carotid atherosclerotic disease, and others. Those factors may be more relevant to the stroke risk analysis than sinus rhythm itself after ablation procedure. In the LAAOS III trial, it was clearly demonstrated that the left atrial appendage closure significantly lowered the risk of ischemic stroke or systemic embolism (16). However, the majority of LAAOS III patients (around 70%) did not undergo AF ablation procedure.

### 4.1 Limitations

The main limitation of the study is its retrospective nature which bears inherent drawbacks, such as selection bias, missing data, and others. Different types of surgery (valve surgery, CABG, etc.) were included into the study representing a real-world clinical practice. Different surgeons performed surgical ablation, but the distribution of patients who received either mode of ablation is equal among operating surgeons. The decision to proceed with either energy mode was made by the operating surgeon. The use of Holter monitoring declined over time and its availability in each year is shown in Supplementary Table S2. Exit and entrance block of the pulmonary veins and left atrium wall were assessed only in early series of patients. Considering the limitations of the study, the association of CE approach with better clinical outcomes should be further investigated in randomized studies. With all above-mentioned limitations, the study represents one of the largest comparative studies on energy modes used in concomitant biatrial ablation, focusing on patients with non-paroxysmal atrial fibrillation.

### 4.2 Conclusions

In the setting of concomitant biatrial ablation for non-paroxysmal AF, combined bipolar radiofrequency and cryoablation showed better results with regards to freedom from atrial arrhythmias, arrhythmia-related hospital readmissions and incidence of ischemic strokes compared to cryoablation alone.

## Data Availability

The original contributions presented in the study are included in the article/Supplementary Material, further inquiries can be directed to the corresponding author/s.
