# Supplementary material for: Concomitant ablation for non-paroxysmal atrial fibrillation: combined energy versus cryoablation alone
Source: Front Cardiovasc Med. 2024 Sep 18;11:1448523. doi: 10.3389/fcvm.2024.1448523 (PMC11444986; doi:10.3389/fcvm.2024.1448523)
Supplement: Supplementary file 1 [file Table1.docx]

Supplementary Material

# Supplementary Tables

## Table S1

Pre- and perioperative data before propensity score matching

| Characteristic | Cryoablation alone; N=262 | Combined energy ablation; N=191 | p value | ASD | Cryoablation alone; N=157 | Combined energy ablation; N=157 | *p* value | *ASD* |
| --- | --- | --- | --- | --- | --- | --- | --- | --- |
| Age, years | 61 (54, 65) | 56 (49, 63) | <0.001 | 0.402 | 59 (50, 64) | 57 (50, 64) | 0.371 | 0.089 |
| Sex, male | 139 (53%) | 118 (62%) | 0.131 | 0.079 | 90 (57%) | 92 (59%) | 0.820 | 0.013 |
| Atrial fibrillation duration, years | 3.0 (1.0, 6.0) | 2.0 (1.0, 5.0) | 0.183 | 0.161 | 3.0 (1.0, 6.0) | 3.0 (1.0, 6.0) | 0.400 | 0.041 |
| Left ventricular ejection fraction, % | 56 (50, 63) | 57 (49, 63) | 0.663 | 0.054 | 57 (51, 63) | 58 (50, 64) | 0.713 | 0.035 |
| Left atrial size ^a^, cm | 6.60 (6.20, 7.25) | 6.60 (6.10, 7.23) | 0.520 | 0.111 | 6.60 (6.20, 7.30) | 6.60 (6.10, 7.30) | 0.891 | 0.026 |
| Previous catheter ablation | 6 (2.3%) | 2 (1.1%) | 0.482 | 0.012 | 3 (1.9%) | 2 (1.3%) | 0.662 | 0.006 |
| Previous myocardial infarction | 19 (7.2%) | 11 (5.8%) | 0.742 | 0.013 | 14 (8.9%) | 10 (6.4%) | 0.421 | 0.025 |
| Body mass index | 28.5 (25.1, 32.4) | 28.2 (25.1, 31.4) | 0.160 | 0.141 | 28.0 (24.1, 32.0) | 28.4 (25.5, 31.6) | 0.781 | 0.03 |
| Hypertension | 164 (62%) | 81 (43%) | 0.002 | 0.158 | 77 (49%) | 76 (48%) | 0.901 | 0.006 |
| Diabetes mellitus | 39 (15%) | 19 (10%) | 0.230 | 0.046 | 21 (13%) | 18 (11%) | 0.590 | 0.019 |
| Degenerative heart valve disease | 117 (44%) | 55 (29%) | 0.003 | 0.152 | 54 (34%) | 48 (31%) | 0.450 | 0.038 |
| Rheumatic heart valve disease | 112 (43%) | 118 (62%) | <0.001 | 0.193 | 88 (56%) | 96 (61%) | 0.312 | 0.051 |
| Ischemic heart disease | 46 (17%) | 28 (15%) | 0.320 | 0.046 | 33 (21%) | 30 (19%) | 0.702 | 0.019 |
| Hypertrophic obstructive cardiomyopathy | 12 (4.5%) | 6 (3.2%) | 0.711 | 0.012 | 5 (3.2%) | 6 (3.8%) | 0.76 | 0.006 |
| Infective endocarditis | 9 (5.3%) | 10 (4%) | 0.732 | 0.013 | 8 (5.1%) | 6 (3.8%) | 0.603 | 0.013 |
| Peripheral vascular disease ^b^ | 85 (32%) | 49 (26%) | 0.251 | 0.057 | 41 (26%) | 41 (26%) | >0.99 | 0 |
| Chronic obstructive pulmonary disease | 23 (8.7%) | 10 (5.3%) | 0.074 | 0.051 | 9 (5.7%) | 7 (4.5%) | 0.621 | 0.013 |
| Previous stroke | 29 (11%) | 11 (5.8%) | 0.121 | 0.049 | 11 (7.0%) | 9 (5.7%) | 0.643 | 0.013 |
| Previous transient ischemic attack | 4 (1.5%) | 2 (1.1%) | >0.99 | 0.004 | 3 (1.9%) | 1 (0.6%) | 0.340 | 0.013 |
| Time in operating room, min | 225 (180, 290) | 270 (220, 328) | <0.001 | 0.544 | 258 (210, 315) | 253 (220, 319) | 0.600 | 0.009 |
| Cross-clamp time | 90 (73, 113) | 107 (88, 135) | <0.001 | 0.492 | 100 (83, 125) | 107 (87, 129) | 0.301 | 0.041 |
| Mitral valve replacement | 125 (47%) | 112 (59%) | 0.004 | 0.147 | 86 (55%) | 95 (61%) | 0.312 | 0.057 |
| Aortic valve replacement | 55 (21%) | 42 (22%) | >0.99 | 0 | 31 (20%) | 33 (21%) | 0.771 | 0.013 |
| Coronary artery bypass grafting | 46 (17%) | 28 (15%) | 0.920 | 0.009 | 21 (13%) | 26 (17%) | 0.462 | 0.032 |
| Atrial tachyarrhythmia recurrence in hospital | 120 (45%) | 60 (32%) | 0.006 | 0.138 | 69 (44%) | 53 (34%) | 0.069 | 0.102 |
| Electrical cardioversion | 63 (24%) | 41 (22%) | 0.981 | 0.006 | 34 (22%) | 38 (24%) | 0.626 | 0.025 |
| Hospital mortality | 6 (2.3%) | 6 (3.2%) | 0.532 | 0.01 | 3 (1.9%) | 5 (3.2%) | 0.481 | 0.013 |
| Perioperative myocardial infarction | 4 (1.5%) | 2 (1.1%) | >0.99 | 0.004 | 3 (1.9%) | 2 (1.3%) | 0.663 | 0.006 |
| In-hospital stroke | 5 (1.9%) | 2 (1.1%) | 0.712 | 0.008 | 2 (1.3%) | 1 (0.6%) | 0.572 | 0.006 |
| Permanent pacemaker implantation in hospital | 19 (7.2%) | 21 (11%) | 0.301 | 0.035 | 11 (7%) | 17 (11%) | 0.263 | 0.038 |
| Atrial tachyarrhythmias at discharge | 49 (19%) | 31 (16.1%) | 0.523 | 0.03 | 31 (20% | 27 (17%) | 0.583 | 0.027 |
| Left ventricular ejection fraction at discharge, % | 56 (50, 61) | 57 (49, 65) | 0.055 | 0.165 | 56 (50, 61) | 58 (49, 65) | 0.141 | 0.146 |

Continuous variables are presented as median with interquartile range. Categorical data are expressed as absolute numbers and relative frequencies. ASD, absolute standardized difference.

^a^ long axis view on transthoracic echocardiography

^b^ hemodynamically significant

## Table S2

Use of prolonged monitoring at each year

| Year | 1 | 2 | 3 | 4 | 5 | 6 | 7 |
| --- | --- | --- | --- | --- | --- | --- | --- |
| Percentage of available Holter data | 88% | 93% | 70% | 71% | 71% | 83% | 95% |
| Ratio of available Holter data to total number of patients available to follow-up | 303/344 | 269/288 | 161/230 | 120/168 | 97/137 | 88/106 | 69/73 |

The availability of Holter monitoring declined over time mainly because of poor patient adherence to recommendations in the absence of arrhythmia symptoms. Notably, almost all patients had ECGs performed several times each year and in patients with lacking Holter data and no ATA, there was sinus rhythm only registered on ECG.
